# H_2_S-Enriched Flush out Does Not Increase Donor Organ Quality in a Porcine Kidney Perfusion Model

**DOI:** 10.3390/antiox12030749

**Published:** 2023-03-19

**Authors:** Hanno Maassen, Leonie H. Venema, Marc G. Weiss, Tobias M. Huijink, H. Sijbrand Hofker, Anna K. Keller, Tom E. Mollnes, Marco Eijken, Søren E. Pischke, Bente Jespersen, Harry van Goor, Henri G. D. Leuvenink

**Affiliations:** 1 Department of Pathology and Medical Biology, University Medical Center Groningen, University of Groningen, 9713 GZ Groningen, The Netherlands; h.maassen@umcg.nl (H.M.);; 2 Department of Surgery, University Medical Center Groningen, University of Groningen, 9713 GZ Groningen, The Netherlands; 3 Department of Renal Medicine, Aarhus University Hospital, 8200 Aarhus, Denmark; 4 Department of Urology, Aarhus University Hospital, 8200 Aarhus, Denmark; 5 Department of Immunology, Oslo University Hospital, University of Oslo, 0372 Oslo, Norway; 6 Institute for Clinical Medicine, University of Oslo, 0372 Oslo, Norway; 7 Research Laboratory, Norland Hospital, 8005 Bodø, Norway; 8 Centre of Molecular Inflammation Research, Norwegian University of Science and Technology, 7034 Trondheim, Norway; 9 Department of Clinical Immunology, Aarhus University Hospital, 8200 Aarhus, Denmark; 10 Department of Anesthesiology, Oslo University Hospital-Rikshospitalet, University of Oslo, 0372 Oslo, Norway

**Keywords:** kidney graft preservation, ex vivo kidney perfusion, transplantation, hydrogen sulphide, H_2_S, ischemia reperfusion injury, brain death, cytokines

## Abstract

Kidney extraction time has a detrimental effect on post-transplantation outcome. This study aims to improve the flush-out and potentially decrease ischemic injury by the addition of hydrogen sulphide (H_2_S) to the flush medium. Porcine kidneys (*n* = 22) were extracted during organ recovery surgery. Pigs underwent brain death induction or a Sham operation, resulting in four groups: donation after brain death (DBD) control, DBD H_2_S, non-DBD control, and non-DBD H_2_S. Directly after the abdominal flush, kidneys were extracted and flushed with or without H_2_S and stored for 13 h via static cold storage (SCS) +/− H_2_S before reperfusion on normothermic machine perfusion. Pro-inflammatory cytokines IL-1b and IL-8 were significantly lower in H_2_S treated DBD kidneys during NMP (*p* = 0.03). The non-DBD kidneys show superiority in renal function (creatinine clearance and FENa) compared to the DBD control group (*p* = 0.03 and *p* = 0.004). No differences were seen in perfusion parameters, injury markers and histological appearance. We found an overall trend of better renal function in the non-DBD kidneys compared to the DBD kidneys. The addition of H_2_S during the flush out and SCS resulted in a reduction in pro-inflammatory cytokines without affecting renal function or injury markers.

## 1. Introduction 

Worldwide, the majority of donor kidneys used for transplantation are derived from deceased brain dead (DBD) donors. Brain death is the potential end result of increased intracranial pressure often caused by cerebral haemorrhage or trauma. When the intracranial pressure exceeds the systemic blood pressure, the subsequent ischemia of the brain results in a release of vast amounts of catecholamines, triggering a cascade of cytokine release to the circulation [1]. The inflammatory response, evoked by brain death, is known to induce organ injury in the donor [2]. In addition to the injurious events in brain dead donors, several unavoidable ischemic episodes occur during kidney transplantation. Extraction time, and cold and warm ischemic time all have a detrimental effect on post-transplantation outcome in kidney transplantation [3,4,5,6,7]. Kidney allografts from brain dead donors are therefore at a higher risk for graft deterioration compared to kidneys from living donors [8,9,10]. To improve the quality of kidneys derived from DBD donors, one could intervene during the ICU phase, but this brings ethical and logistical issues [11]. A more isolated approach, i.e., treatment of the organ during or after retrieval allows target treatment and might be better suitable. 

Hydrogen sulphide (H_2_S) is a gasotransmitter known for its anti-inflammatory capacities [12]. H_2_S has antioxidant properties [13], and can induce a hypometabolic state in isolated perfused organs [14]. These qualities could result in the protection of kidneys during the first flush and subsequent preservation time. H_2_S may counteract the inflammatory response induced by brain death and prevent ischemic injury. Kidneys warm up during the procurement surgery, and the capacity of H_2_S to reduce the metabolic rate could further diminish injury by reducing its metabolism. H_2_S-enriched flush-out and H_2_S-enriched cold storage showed beneficial effects in a rat renal transplantation model [15]. However, experimental data on larger mammals, such as pigs and sheep, failed to show hypometabolic action when H_2_S was administered systemically [16,17]. An isolated administration of H_2_S might be more effective. The potential therapeutic properties of H_2_S have not been tested in large brain-dead mammals so far.

The present study aims to improve the flush-out and preservation procedure by decreasing the inflammatory response and ischemic injury through addition of H_2_S to the preservation solution in kidneys from brain dead and non-brain-dead pigs. We hypothesized that H_2_S decreases the cytokine release and minimizes the amount of reactive oxygen species (ROS) formed during ischemia, thereby reducing inflammation and ischemia-reperfusion injury. Mitochondrial ROS are important early drivers of ischemia-reperfusion injury, formed by the primary driver succinate, which accumulates during ischemia [18]. In this study, normothermic machine perfusion (NMP) was used as a reperfusion model to test short-term post-preservation renal function [19]. Analysis included metabolic parameters, renal function, injury and oxidative stress markers, cytokines release, and complement activation. 

## 2. Methods

### 2.1. Study Design 

**Figure 1:**
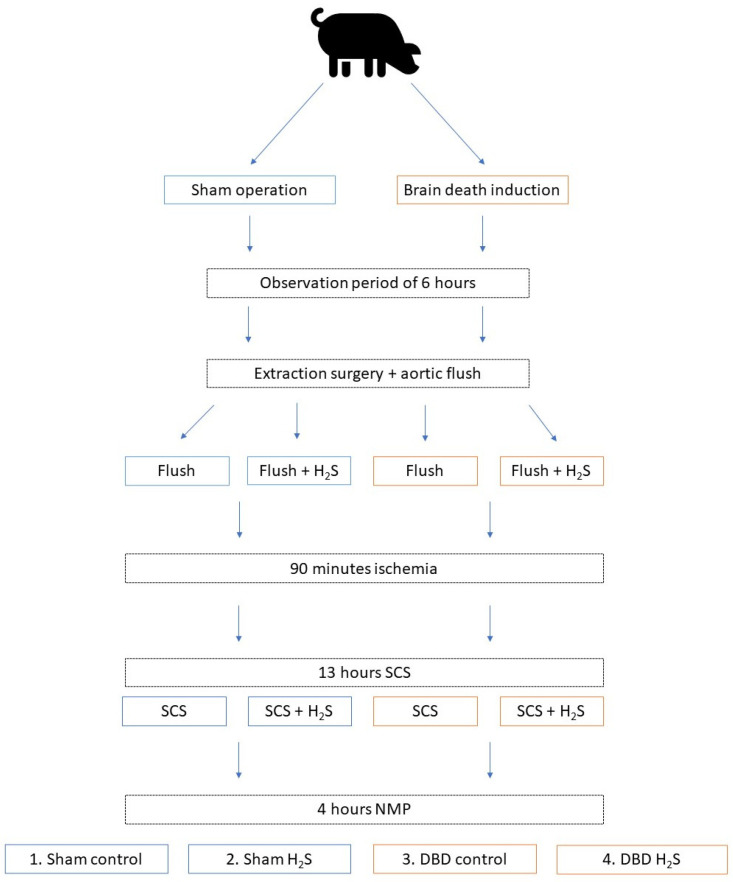
Experimental overview. Group 1 Sham control, group 2 Sham + H_2_S treatment, group 3 DBD control, group 4 DBD + H_2_S treatment. Abbreviations: DBD = donation after brain death. H_2_S = hydrogen sulphide. NMP = normothermic machine perfusion. SCS = static cold storage.

In order to minimize the number of laboratory animals needed for this study, kidneys were included as a sub-study from another larger study from our group. We retrieved kidneys from 6 brain dead and 5 Sham control pigs. The overview of the experimental setup is displayed in Figure 1. After brain death induction or the Sham control procedure, pigs were observed for six hours before extraction surgery. During procurement, the abdominal organs were flushed with 4 L of cold University of Wisconsin-static cold storage solution (UW-SCS) before extraction. For the present study, kidneys were flushed with 500 mL cold UW-SCS with or without H_2_S (1 mmol/L) and stored at room temperature for 90 min to simulate the prolonged ischemic injury following a human multi-organ donor procedure. All kidneys were cold stored for 13 h in either UW-SCS supplemented with H_2_S (1 mmol/L) in the H_2_S groups or UW-SCS without H_2_S in the control groups. After the storage time, the kidneys were flushed with saline (0.9%, 60 mL) before NMP was performed for 4 h. The experimental setup resulted in four groups: Sham control, Sham H_2_S, DBD control, and DBD H_2_S.

### 2.2. Ethics and Animals

A total of 11 female Danish landrace pigs purchased from the same breeder, weighing on average 63.5 kg (range 59.3–68 kg), were used for organ extraction. As previously stated, the pigs were simultaneously used for several research projects to minimize the number of animals used for experiments. The small intestine was used for a luminal preservation experiment. Biopsies from the liver, pancreas, m. psoas, lungs, and heart were used to analyse the effect of brain death on different organ systems. For this project, the kidneys were used and assessed on NMP. All animal care and procedures were conducted in accordance with guidelines by the European Union (directive 2010/63/EU) and local regulations. The study was approved by the Danish Animal Experimentation Council (reference-number 2019-15-0201-00157). All personnel involved had Federation of European Laboratory Animal Science Associations licenses or equivalent.

### 2.3. Anaesthesia and Induction of Brain Death 

Pigs were sedated prior to anaesthesia with an intramuscular injection of Zoletil-mix (Zoletil 50 vet. (125 mg Tiletamin, 125 mg Zolazepam), 125 mg Xylazin, 125 mg Ketamin, 25 mg Butorphanol). An 18G intravenous catheter was placed in each ear and 50–100 mg Propofol was administered i.v. prior to intubation if the sedation with the Zolet-mix was not sufficient for the intubation procedure. Anaesthesia was maintained using Propofol (8 mg/kg/h) (Fresenius Kabi, Bad Homburg von der Höhe, Germany) and Fentanyl (10 µg/kg/h) (B Braun, Melsungen, Germany). Animals were placed in supine position and were intubated. After intubation, a pressure sensor was placed in the coronary artery and a urine catheter was placed in the bladder. Ventilation was achieved with a tidal volume between 8–10 mL/kg and regulated to a PaCO_2_ between 5.5–6.5 kPa. An arterial sheath was placed in the left common carotid artery and two sheaths were placed in the jugular veins, one on each side. A 14Fr urinary catheter (Teleflex, Wayne, PA, USA) was placed and connected to a two-chamber collection bag (Unomedical, Lejre, Denmark), to monitor urine production. After an infusion of 1000 mL of ringer’s acetate, 400 mL of blood was drawn from the pigs via one of the jugular sheaths, used for perfusion during NMP. 

Induction of brain death was performed according to a previously established model [20]. The animal was turned to a prone position and a 10 cm incision was made along the sagittal suture. The skin and periosteum were retracted using a self-retaining retractor. Intracranial pressure monitoring was established in all animals using a RALK hand drill with a 5 mm drill bit, a CH5 bolt, and a NEUROVENT-PTO catheter (Raumedic, Helmbrechts, Germany). A 20 mm Hudson hand drill and back-biting forceps were used for the brain death groups (groups 3 and 4) to make a burr hole where a CH22 urine catheter (Unomedical, Lejre, Denmark) was placed. The balloon of the urinary catheter was filled with saline, at a rate of 1 mL/min for 10 min, followed by 0.5 mL/min for an additional 10 min, and 0.25 mL/min, until a persistent negative cranial perfusion pressure confirmed brain death. Cranial perfusion pressure was defined as the mean arterial pressure minus the intracranial pressure. Following a negative cranial perfusion pressure, an additional 10 mL was injected into the balloon as a bolus to ensure brain death. 

### 2.4. Organ Retrieval and Organ Flush

Before the organ retrieval surgery, pigs were turned to a supine position. Via a midline laparotomy, the abdomen was opened together with the peritoneum. Kidneys were dissected free of the surrounding tissue, together with the ureter, aorta, and vena cava. Kidney biopsies and urine samples were taken. Kidneys were then left in place for further preparation of the remaining organs and vasculature. An amount of 500 IE/kg of heparin (LEO Pharma, Ballerup, Denmark) was administered, and after 3 min, a St. Thomas cannula (Medtronic, Dublin, Ireland) was placed in the ascending aorta. The distal abdominal aorta and vena cava inferior were ligated, and the abdominal aorta was cannulated using a 14F cannula (Bridge to life, London, UK). While the abdominal cannulation was performed, an additional 600 mL of blood was removed via the St. Thomas cannula prior to the cold St. Thomas I solution infusion. 

The ascending aorta was cross clamped, 1 L of cold St. Thomas I solution was infused under pressure through the St. Thomas cannula (Medtronic, Dublin, Ireland), and the inferior vena cava was transected above the diaphragm. The abdominal aorta was cross clamped above the liver, 4 L of cold Belzer UW-SCS (Bridge to life, London, England) was infused under pressure via the cannulation of the abdominal aorta. The inferior vena cava was transected proximally to the ligature, preventing the venous return from the legs. Crushed ice (50 mg/mL glucose saline) (Fresenius Kabi, Bad Homburg von der Höhe, Germany) was placed around all transplantable organs. Next, the kidneys were extracted after the other organs were removed. Left and right kidneys were structurally assigned to one group, eliminating any effect of anatomical difference and extraction time on outcomes. One kidney was flushed at the back table with 500 mL of cold UW-SCS, and the other kidney was flushed with UW-SCS supplemented with 1 mmol of H_2_S. To induce additional ischemic injury, explanted kidneys were placed at room temperature for 90 min in the flush fluid (reaching 20 degrees Celsius at the end). After 90 min, the surrounding solution was replaced with cold fresh UW-SCS (+/− H_2_S), and the kidneys were stored for 13 h. 

### 2.5. Normothermic Machine Perfusion

NMP was simultaneously performed on each kidney pair to ensure similar ischemic times. The NMP procedure was performed as described earlier [21]. Briefly, a sinusoidal pulse was applied with a mean pressure of 75 mmHg for 4 h using a centrifugal pump (Deltastream DP3^®^, MEDOS Medizintechnik AG, Heilbronn, Germany) controlled by in-house developed software (Sophistikate^®^, Labview, National Instruments, Austin, TX, USA). The temperature was regulated using a Jubalo water heating system and was set at 37 degrees Celsius. An integrated heat exchanger (HILITE 1000^®^, MEDOS Medizintechnik AG, Heilbronn, Germany) was built into the oxygenator. A clamp-on flow sensor was used to measure flow (ME7PXL clamp^®^, Transonic Systems, Inc., Ithaca, NY, USA). The pressure sensor used was a Truewave^®^ disposable pressure transducer (Edwards Lifesciences, Irvine, CA, USA). As perfusion medium, 170 mL red blood cells (RBCs), 250 mL 5% human albumin, 5 mL 8.4% Sodium bicarbonate, 6 mL 5% glucose, 3 mL 10% calcium gluconate, 5 mL 1000/200 mg Co-Amoxiclav, 31 mg creatinine, 5 IU insulin, and 1 mL (5 mg/2 mL) verapamil was used. Carbogen (95% O_2_ and 5% CO_2_) was supplied via the oxygenator at a flow rate of 500 mL/min. Continuous infusion of verapamil (0.25 mg/h) was supplied via the arterial tube. A 60 mg bolus of Co-Amoxiclav (1000/200 mg) was administered every hour. Perfusate and urine samples were taken serially, together with blood gas samples. 

### 2.6. Flush Samples and Biopsies 

Flush samples were collected after extraction and flush of the kidney, after cold storage, and the saline flush, and just before NMP. Tissue was collected at the same time points via a punch-biopsy (4 mm). The punch-biopsy was divided into three pieces, one stored in formalin, one in liquid nitrogen, and one in a buffer for ATP analysis (0.372 g EDTA (0.744 g/L) in 130 mL H_2_O and NaOH (pH 10.9) + 370 mL 96% ethanol). After NMP, two 1 cm^3^ biopsies were additionally taken and snap-frozen in liquid nitrogen. Periodic acid-Schiff (PAS) staining was performed on the paraffin-embedded biopsies, which were analysed by an experienced pathologist.

### 2.7. Cytokines and Complement Activation Products

Cytokine and complement activation products were determined in the NMP perfusate, urine produced during NMP, SCS fluid, and flush-out fluids. Fluids were stored in EDTA tubes for the complement analysis and in standard Eppendorf tubes for the cytokine levels. Terminal complement complex (TCC) and C3a were measured using in-house ELISA, as described in detail previously [22,23]. Tumor necrosis factor (TNF), IL-1b, IL-6, IL-8, and IL-10 were measured using commercially available ELISA and multiplex assays as described previously described [24]. 

### 2.8. Renal Function and Injury Markers

During NMP, the flow was registered every minute. The sampling of perfusate and urine, and blood gas analysis of the perfusate, were performed immediately after the start of perfusion, after 15 min, 30 min, 60 min, and every following hour. Perfusate and urine samples were stored on ice until centrifuging at 1000× *g* for 12 min at 4 degrees Celsius. Next, supernatants were aliquoted and stored at −80 degrees Celsius until further analysis.

Renal metabolism and function were calculated with oxygen consumption, metabolic coupling of sodium transport by ATPase in tubular epithelial cells, fractional sodium excretion (FENa), and creatinine clearance using the previously described formulas [25].

Creatinine, sodium, potassium, and protein concentration in both the perfusate and urine samples were analysed via routine clinical procedures at the University Medical Center Groningen (UMCG) clinical chemistry lab. ATP levels were measured as previously described [26] using the ATP Bioluminescence Assay Kit CLS II (Roche Diagnostics, Mannheim, Germany) according to a standardized protocol and expressed relative to the protein concentration (Pierce^TM^ BCA Protein Assay Kit, Rockford, IL, USA). Neutrophil gelatinase-associated lipocalin (NGAL) levels were measured in urine using ELISA (pig NGAL ELISA kit 004, BioPorto, Needham, MA, USA). Lipid peroxidation product malondialdehyde (MDA) was measured in tissue homogenates as described previously [27]. BAX/BCL-2 ratios were measured using Real-Time PCR as described previously [28], fragments of these genes were amplified with the primer sets outlined in Table 1.

### 2.9. Statistics

All data are expressed as median with interquartile range unless stated otherwise. The area under the curve was calculated in data with multiple time points during NMP. Statistical analysis on values during NMP was performed on the calculated AUC during the whole course of NMP. The effect of H_2_S treatment was evaluated in both the DBD and Sham kidneys. In addition, the effect of donation type was evaluated by comparing the DBD control group with the Sham control group. Differences between the DBD control and DBD H_2_S group and differences between the Sham control and Sham H_2_S group were calculated using Wilcoxon matched-pairs signed-rank tests. Differences between the DBD control and Sham control group were calculated using Mann–Whitney U tests. A *p* value <0.05 was considered statistically significant. Data were analysed with Graphpad PRISM 8.4.2 (GraphPad, San Diego, CA, USA).

## 3. Results 

### 3.1. Baseline Characteristics

Baseline characteristics of the animals and kidneys are shown in Table 2. No significant differences were seen between the Sham (*n* = 10) and the DBD (*n* = 12) kidneys, except for urine production. As expected, DBD pigs produced significantly more urine compared to the Sham pigs during the observation period (4184 mL vs. 963 mL, *p* < 0.001).

### 3.2. Cytokine Release during Preservation and NMP

**Figure 2:**
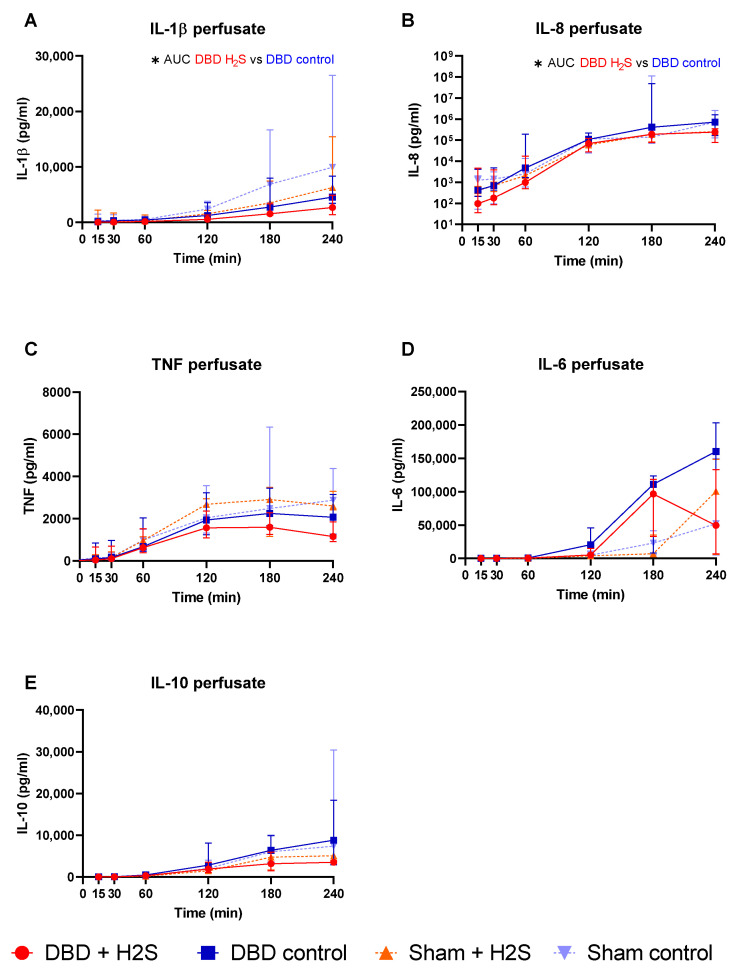
Cytokine release. (**A**–**E**): respectively IL-1b, IL-8, TNF IL-6, and IL-10 levels (pg/mL) in the perfusate during NMP. Data presented as median + interquartile range. * *p* < 0.05. Abbreviations: AUC = area under the curve. TNF = tumor necrosis factor. NMP = normothermic machine perfusion.

The addition of H_2_S in the flush-out solution and cold storage solution resulted in lower IL-1β in the H_2_S-treated DBD kidneys compared to the DBD control kidneys (*p* = 0.03) (Figure 2A). H_2_S treatment also decreased IL-8 levels in the perfusate during NMP in the DBD kidneys (*p* = 0.03) (Figure 2B). H_2_S treatment in the Sham group did not alter IL-1β or IL-8 levels. No significant effects of H_2_S or donor type were seen for TNF, IL-6, and IL-10 during NMP (Figure 2C–E). 

### 3.3. Complement Activation during Preservation and NMP 

**Figure 3:**
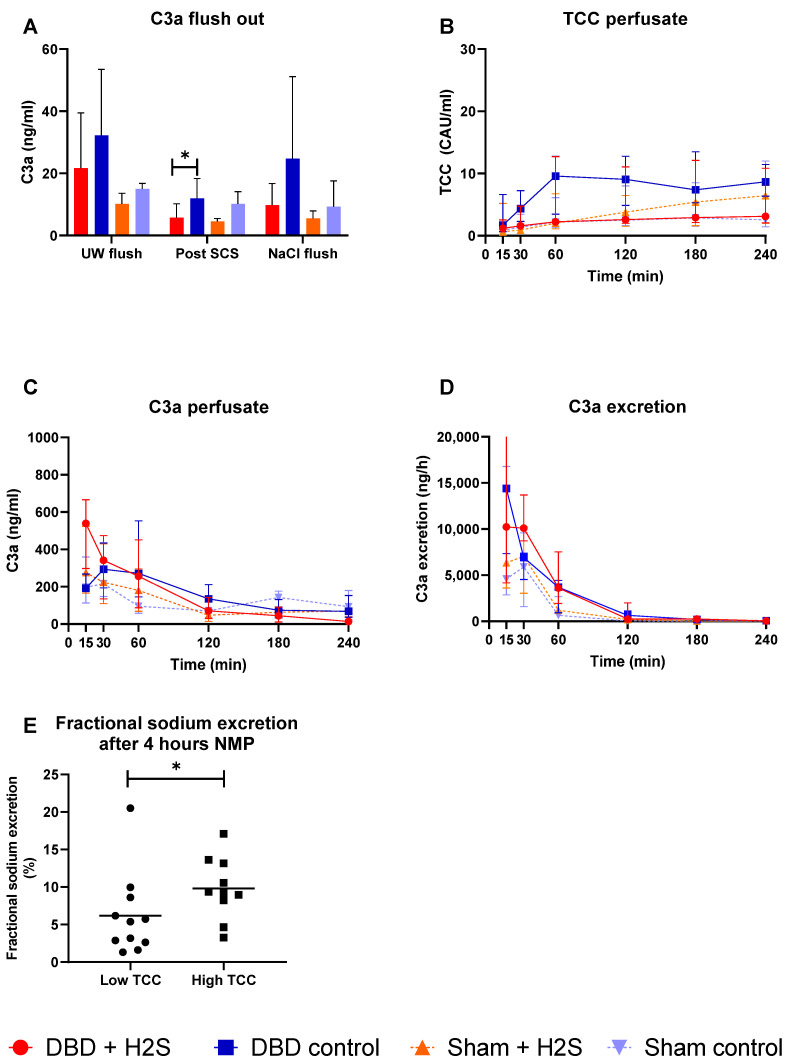
Complement activation. (**A**): C3a levels after the flushes and after SCS (ng/mL). (**B**): TCC in the perfusate during NMP (CAU/mL). (**C**): C3a in the perfusate during NMP (ng/mL). (**D**): C3a excretion in the urine (ng/h). (**E**): Fractional sodium excretion after 4 h NMP in the lowest TCC kidneys and the highest TCC kidneys. Data presented as median + interquartile range. * *p* < 0.05. Abbreviations: TCC = terminal complement complex. NMP = normothermic machine perfusion. SCS = Static cold storage.

Complement activation was measured in the UW-flush after nephrectomy and in the NaCl-flush before NMP, after SCS, and during different time points of NMP. H_2_S treatment in the DBD kidneys decreased the C3a levels in the SCS fluid (*p* = 0.03) (Figure 3A). No effect of H_2_S treatment was found in the Sham kidneys. TCC levels in the perfusate increased in all groups during NMP (Figure 3B). C3a levels in both the perfusate (Figure 3C) and urine (Figure 3D) started higher and decreased during NMP independent of donation type. To investigate the impact of higher complement activation on renal function, we dichotomized all kidneys in a group with the lowest and the highest TCC values. FENa was significantly lower (better tubular function) in the low TCC group compared to the high TCC group (*p* = 0.036) (Figure 3E). 

### 3.4. Metabolic Activity and ROS

**Figure 4:**
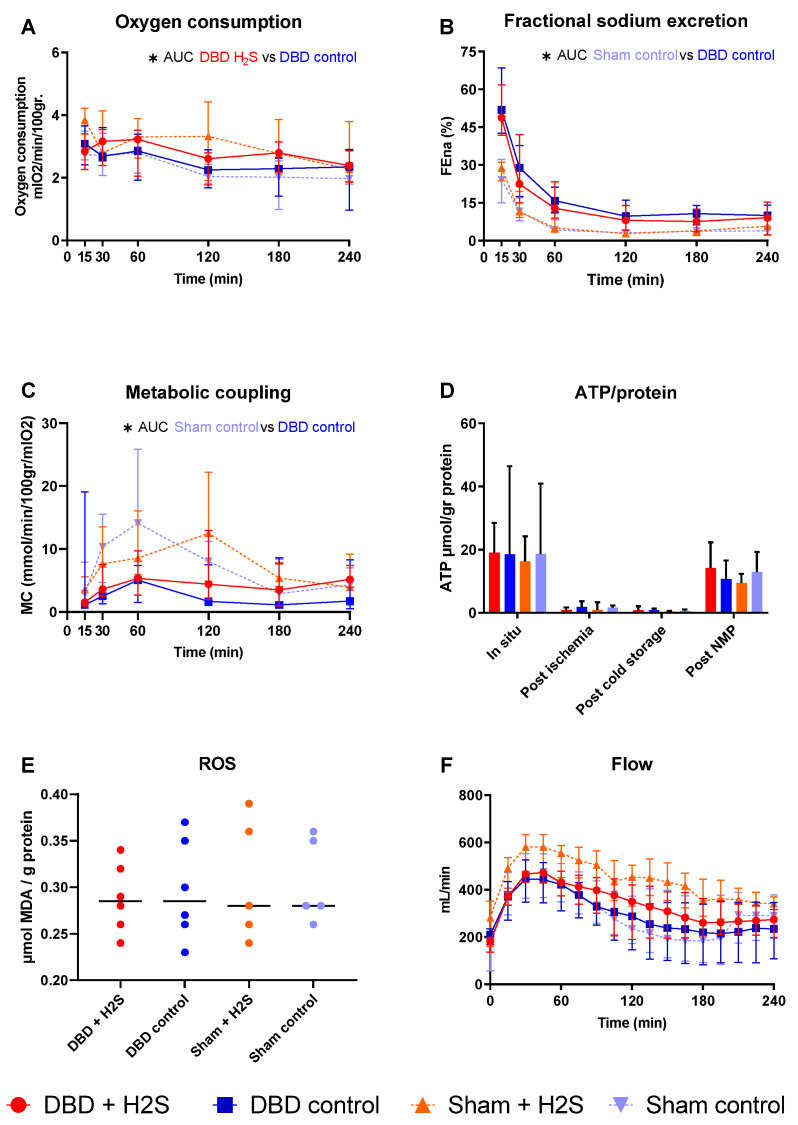
Metabolic activity and ROS. (**A**): Oxygen consumption (mlO_2_/min/100 gr). (**B**): Fractional sodium excretion (%). (**C**): Metabolic coupling (mmol/min/100 gr/mlO_2_). (**D**): ATP values during preservation and after NMP (µmol/gr protein). (**E**): MDA after NMP, measured in tissue (µmol MDA/gram protein). (**F**): Arterial blood flow during NMP (mL/min). Data presented as median + interquartile range. * *p* < 0.05. Abbreviations: AUC = area under the curve. MDA = malondialdehyde.

After SCS, kidneys were perfused by NMP for 4 h using an RBC-based perfusate to assess the metabolic activity of the kidneys (Figure 4). There was a significantly higher oxygen consumption in the H_2_S-treated DBD kidneys compared to the control DBD kidneys (*p* = 0.03) (Figure 4A). H_2_S treatment did not impact other metabolic or ROS parameters in the DBD groups. In the Sham groups, H_2_S did not alter oxygen consumption. FENa was calculated to estimate tubular function (Figure 4B). Metabolic coupling, providing information about how much oxygen is used for sodium transport, was also not affected by H_2_S treatment (Figure 4C). ATP levels were measured at four different time points, and the kidneys had the highest ATP values while they were still in situ, just before extraction of the kidneys (Figure 4D). After ischemia and cold storage, ATP values were low and comparable between all groups. NMP increased ATP values in all groups. MDA, indicative of the oxidative injury, showed similar values in all groups after 4 h of NMP (Figure 4E). The arterial blood flow showed an increase in the first hour of perfusion, after which it stabilized, independent of the group (Figure 4F). The type of donor affected the tubular function and metabolic coupling. There was significantly lower FENa in the Sham control kidneys than the DBD control kidneys (*p* = 0.004), and significantly higher metabolic coupling (*p* = 0.03).

### 3.5. Renal Function and Injury Markers 

**Figure 5:**
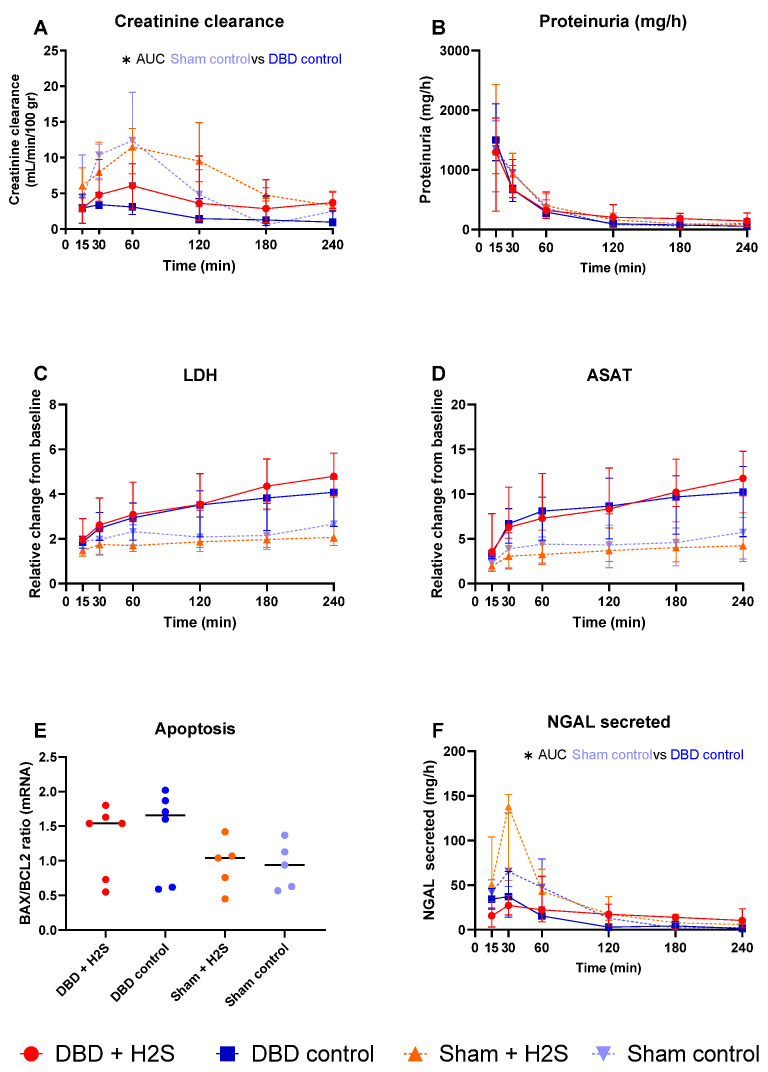
Renal function and injury markers. (**A**): Creatinine clearance (mL/min/100 gr). (**B**): Proteinuria (mg/h). (**C**): LDH (relative change from baseline). (**D**): ASAT (relative change from baseline). (**E**). BAX/BCL2 ratios, measured in tissue after NMP. (**F**): NGAL secretion in urine (mg/h). Data presented as median + interquartile range. * *p* < 0.05. Abbreviations: AUC = area under the curve. LDH = lactate dehydrogenase. ASAT = aspartate aminotransferase. NGAL = urinary neutrophil gelatinase-associated lipocalin. BAX = bcl-2-like protein 4. BCL2 = B-cell lymphoma 2.

**Figure 6:**
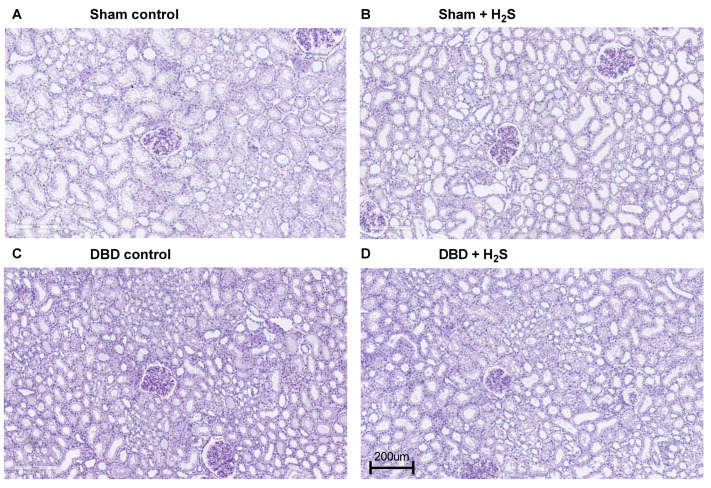
Representative histopathological images.

Creatinine clearance was calculated as an estimation of glomerular filtration (Figure 5A). The H_2_S enriched flush and subsequent cold storage did not alter creatinine clearance in the DBD groups. H_2_S treatment also did not affect creatinine clearance in the Sham kidneys. All kidneys had similar proteinuria during NMP (Figure 5B). Lactate dehydrogenase (LDH) (Figure 5C) and aspartate aminotransferase (ASAT) (Figure 5D) increased over time during NMP. BAX/BCL-2 ratios, indicative of the amount of apoptosis present in the biopsy, showed similar values after NMP, independent of H_2_S treatment or donor type (Figure 5E). The Sham control kidneys showed a higher creatinine clearance compared to the DBD control kidney, indicating an adverse effect of brain death on kidney function (*p* = 0.03) (Figure 5A). NGAL secretion levels were measured in the urine as a marker for renal injury and were significantly higher in the Sham control group vs. the DBD control group (*p* = 0.03) (Figure 5F). Kidneys were histologically examined, we did not find any structural changes in the kidneys. Kidneys were evaluated for glomerular, vascular, and interstitial damage (Figure 6).

## 4. Discussion

The objective of our study was to examine the potential of the addition of H_2_S to the flush-out and cold storage solution, to reduce inflammation and ischemic injury in kidneys retrieved from brain dead donors and non-brain-dead donors. In our approach, the addition of H_2_S reduced levels of the pro-inflammatory cytokines IL-1β and IL-8 during NMP without affecting renal function or renal injury markers. 

Anti-inflammatory characteristics of H_2_S have been studied extensively in vitro, with a suggested role for the attenuation of the nuclear-factor-kappa B (NF-kB) pathway [29] and activation of the protective Keap1/NRF2 signalling pathway. A decrease in cytokine levels due to H_2_S has previously been described in IRI and transplantation models. For example, Sun et al. showed lower levels of TNF and IL-1β after reperfusion in NaHS-enriched UW in a rat heart transplantation model [30]. Additionally, NaHS treatment in a rabbit model of urinary-derived sepsis showed downregulation of pro-inflammatory markers TNF and NF-kB, and upregulation of anti-inflammatory IL-10 [31]. Our data support these findings with a lower level of pro-inflammatory cytokines IL-1β and IL-8 in the perfusate of the H_2_S treated DBD kidneys during NMP. No response to the H_2_S treatment was seen in the Sham kidneys. The lack of impact of H_2_S in the Sham kidneys could not result from higher cytokine levels in the DBD groups, since the Sham group had comparable levels of both IL-1β and IL-8. IL-1β is strongly proinflammatory and is released at the early stages of the immune response to infections or stress [32]. IL-8 is a potent chemoattractant for neutrophils, produced by macrophages [32]. The source of these cytokines might differ between the DBD and Sham pigs, resulting in equal levels of cytokines during NMP, but a stronger effect of H_2_S on the DBD group compared to the Sham group. Brain death and NMP are known to induce a release of cytokines, possibly indicating the different triggers of cytokine release in both groups [33,34]. 

Reperfusion induces activation of the innate immune system, with an essential role in complement activation and cytokine release [35]. The complement system, consisting of three different pathways, the classical, lectin, and alternative pathway, is activated after reperfusion. The lectin pathway has been suggested as a primary role of renal complement activation following ischemia reperfusion injury (IRI) [36]. Brain death has also been shown to affect the innate immune system, probably by the release of alarmins triggering both complement and Toll-like receptors, leading to a systemic cytokine storm affecting distant organs, including the kidneys [37]. Treatment of H_2_S in both DBD and non-DBD donors could identify a possible decrease in injury initiated by the complement system. Complement levels of C3a were lower in the H_2_S treated DBD group after SCS. A similar impact of H_2_S treatment was not seen in the Sham control kidneys. Excessive complement activation can lead to cell lysis and necrosis [38], but the decrease in complement activation by H_2_S was not seen during/after NMP. This might account for the lack of differences in apoptotic markers after NMP. Comparing DBD and Sham kidneys could distinguish between the impact of brain death and ischemia-induced immune system activation. Although our group described an increase in complement activation during different phases of transplantation [39], in this study, no significantly higher activation of complement during NMP in the DBD group compared to the Sham control group were observed. In all groups, TCC showed an increase in the first 2–3 h of reperfusion, whereafter it seemed to stabilize. For C3a, the opposite was the case. C3a showed a higher level at the start of reperfusion with a decrease over time, which can be explained by different activation and turn-over kinetics. There is evidence for complement activation in proximal tubular epithelial cells [40], which might be responsible for the production of TCC over time. The decrease of C3a during NMP might be due to a higher excretion than production during NMP, especially visible in the high secretion values at the start of NMP. When dichotomizing all kidneys in a group with high and low TCC, we showed that the low TCC group had a significantly improved FENa (Figure 4E). The higher FENa in the high TCC group implies that more complement activation might result in worse tubular function in these kidneys, independent of donation type. Treatment of kidneys on NMP with complement inhibitors could lead to improved renal quality prior to transplantation. 

It should be noted that kidneys from all groups showed increased cytokines and TCC during NMP over time. With NMP being increasingly tested for several purposes, including preconditioning, and with clinical implementation around the corner [19], one should contemplate which kidney should be put on NMP and for what reason. Cytokines and complement activation products during NMP might harm rather than improve the kidney before it is transplanted. Especially in living donors, where injury is minimal, the added value of NMP is very limited compared to the risk. On the other hand, NMP could provide a platform and opportunity to counteract or alter the activation of complement and release of cytokines, decreasing possible future injury. Pre-clinical research on this topic shows promising results in decreasing renal injury in bran-dead rats [41].

After the initial paper by Blackstone et al. [42], we and others investigated the hypometabolic capacity of H_2_S. H_2_S can induce a hypometabolic state when administered to small mammals, such as mice. H_2_S competes with O_2_ for binding to cytochrome c oxidase (complex IV) of the electron transport chain in mitochondria. As a result, O_2_ cannot react with H^+^ to form H_2_O, blocking ATP synthase [43], thereby inducing a hypometabolic state. We previously showed that this form of hypometabolism is also conductible in isolated perfused kidneys from large animals otherwise not responsive to H2S-indcued hypometabolism [14]. Impairing the metabolism of the kidney ex vivo during the flush could diminish metabolic activity and decrease organ injury. During NMP, the H_2_S treated kidneys were more metabolically active, displayed by oxygen consumption. These results are in line with our previous experiment where H_2_S treatment resulted in higher oxygen consumption after the hypometabolic effect of H_2_S had passed [14]. In our experimental setup, the addition of H_2_S did not result in a decrease in MDA levels, apoptosis or NGAL levels or better preservation of the renal and tubular function within the time frame studied. Although the induction of brain death highly affected renal function, alterations by H_2_S treatment were not visible. H_2_S therapy has shown beneficial results in counteracting ischemic conditions in previous experiments in small mammals [15,44]. Although promising results are also shown in porcine kidneys regarding ameliorating ischemic injury [45], testing in an extensive transplantation model is still lacking. Although we did not see clear evidence of the efficacy of H_2_S to improve renal function, it cannot be excluded that this is due to the model used. In our model we applied the H_2_S flush at the back table after the initial systemic flush. The application of H_2_S in the first flush could be beneficial, but it would also reach other transplantable organs with unknown effects. The second disadvantage of our setup is the short observation time of 4 h of NMP. NMP is a valuable option to test the early function of organs after retrieval from the donor, as can be observed in the impaired renal function in the DBD group. Longer follow-up in the NMP model is challenging, and therefore, transplantation experiments are needed to examine the long-term effects. The potential direct ischemia reducing characteristics of H_2_S might be limited, and a longer follow-up should elucidate if a decrease in cytokines reduces, for example, fibrosis on the long term. Lastly, the sample size that was used is small, and therefore, the power of the study is limited. 

## 5. Conclusions

The addition of H_2_S in flush-out and storage conditions showed a reduction in inflammatory markers, without affecting renal function or injury markers. At this stage, H_2_S cannot be recommended as an ischemia reducing treatment strategy during a second flush-out after extraction of the kidney. This study may provide a base for future research that focuses on anti-inflammatory and anti-ischemic therapies in more compromised conditions, such as those observed in DBD donor kidneys.

## Figures and Tables

**Table 1:** Primer sequences used for Real-Time PCR.

Gene	Primers	Amplicon Size (bp)	Gene Accession Number
*b-act*	TCTGCGCAAGTTAGGTTTTGTC CGTCCACCGCAAATGCTT	78	AY_550069.1
*Bax* *BCL-2*	CAGACGGTGACCATCTTCGTG TTCTTCCAGATGGTGAGCGAG AATTACCATCGGCGTAGTGCA TCTAGAGCCCTTGACCTCCATCT	58 82	XM_003127290.5 XM_021099593.1

**Table 2:** Overview of the characteristics of the pigs.

In Situ	DBD Kidneys *n* = 12 (6 Control, 6 H_2_S)	Sham Kidneys *n* = 10 (5 Control, 5 H_2_S)	*p*-Value
Weight pigs (kg)	63.5 (3.4)	62 (3.78)	0.689
Time under anaestesia (hours)	11:15 (0:26)	11:08 (0:22)	0.180
Surgery time (hours)	3:06 (0:25)	3:15 (0:17)	0.233
Urine production pigs (mL)	4213 (890)	874 (214.8)	<0.001
**Post Nephrectomy**			
Flush time (min)	18 (7.1)	16.5 (5.7)	0.354
Cold ischemic time (min)	786 (7.3)	785 (11.2)	0.974
Warm ischemic time (min)	1.5 (1.5)	1 (1.1)	0.964
Weight kidneys (gr)	145 (9.5)	143 (7.9)	0.974

Abbreviations: DBD = donation after brain death. Data are presented as median + standard deviation.

## Data Availability

Raw data can be found in Supplementary Material.

## Supplementary Materials

The following supporting information can be downloaded at: https://www.mdpi.com/article/10.3390/antiox12030749/s1.
